# Supplementary material for: Regionalized tissue fluidization is required for epithelial gap closure during insect gastrulation
Source: Nat Commun. 2020 Nov 5;11:5604. doi: 10.1038/s41467-020-19356-x (PMC7645651; doi:10.1038/s41467-020-19356-x)
Supplement: Supplementary file 1 — Supplementary Information [file 41467_2020_19356_MOESM1_ESM.docx]

**Supplementary Figure 1**

**
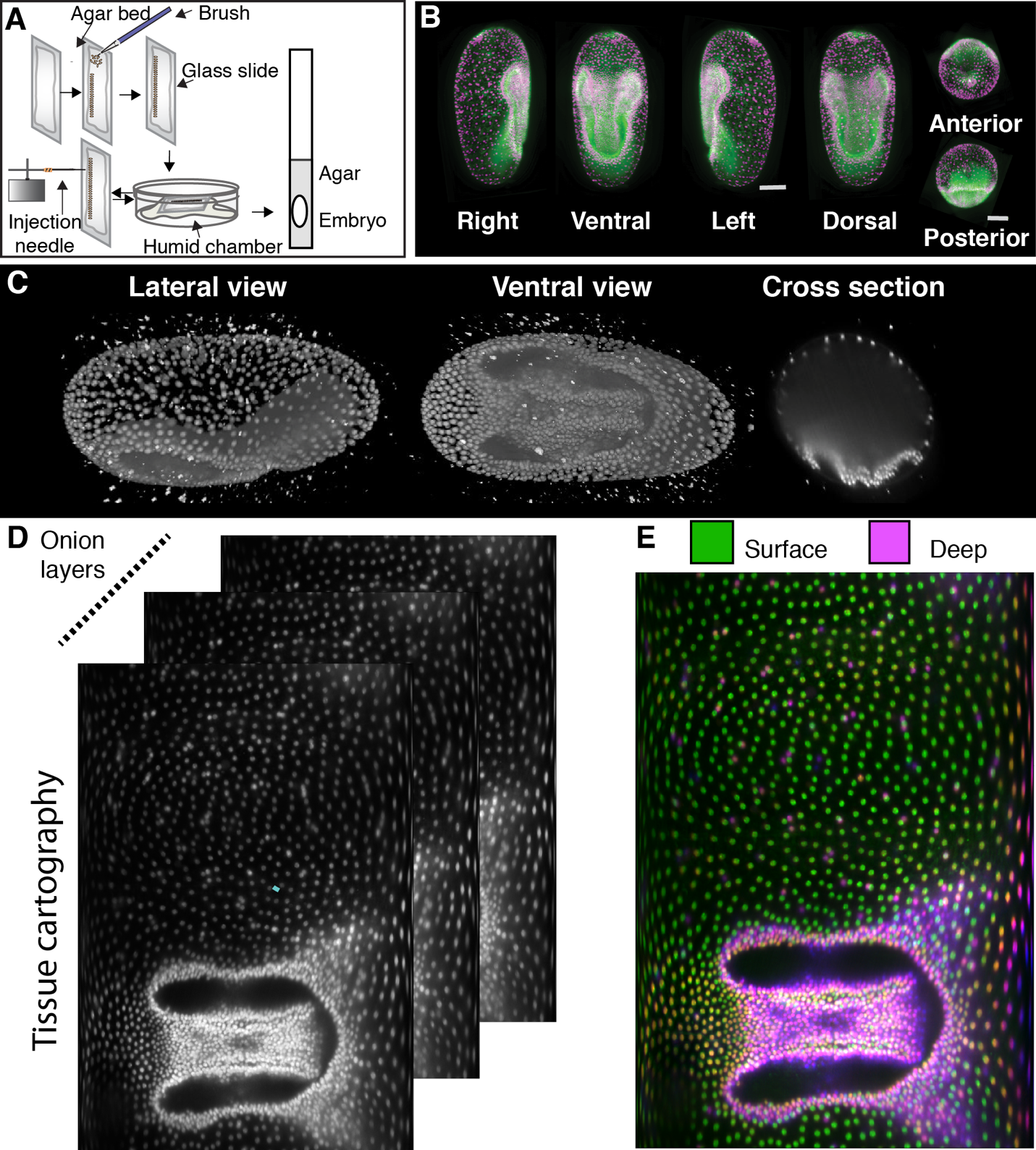
**

**Supplementary Figure 1: *Tribolium* embryo preparation, imaging and image analysis pipeline to study serosa epiboly**

(**A**) Illustration outlines the micro-injection and sample mounting protocol to label *Tribolium* embryos and mount them for light-sheet microscopy.

(**B**) Maximum intensity projections from different views of an embryo injected with *LifeAct-eGFP* and *Histone-RFP* mRNAs. The embryo was imaged from 5 views with light-sheet microscopy. Individual z-stacks (views) were registered and fused based on fluorescent beads scattered in the mounting medium using the Fiji Multi-view Reconstruction plugin (N=1). Scale bar is 50 µm.

(**C**) Histone-eGFP expressing *Tribolium* embryo reconstructed from multi-view SPIM data. The embryo is shown in ventral and lateral 3D renderings and in cross-section. Bright dots surrounding the embryo correspond to the fluorescent beads used for image registration and fusion.

(**D**) Cartographic projections of the embryo shown in (C) with dimensionality reduction from 3D to 2D. Successive, increasingly deeper onion-like layers of the embryo are shown as separate maximum intensity projections.

(**E**) Maximum intensity projection of the different layers shown in (D) color-coded based on depth.

**Supplementary Figure 2**

**
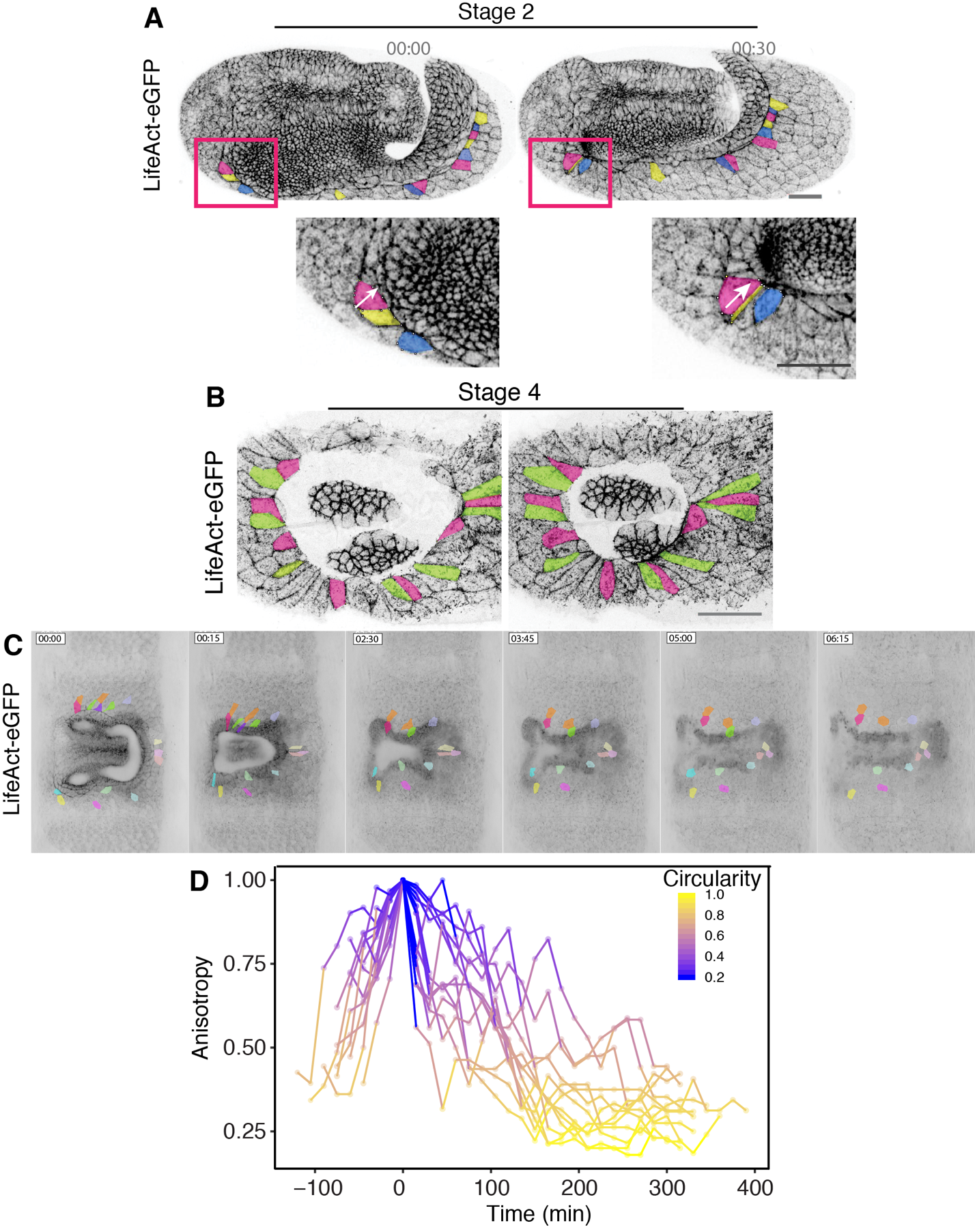
**

**Supplementary Figure 2: Anisotropy of the cells at the leading edge of the serosa increases over time and decreases once they leave the edge**

(**A**) Inverted confocal images of a Life-Act-eGFP expressing *Tribolium* embryo showing selected cells (highlighted with different colors) at the serosal edge at Stage 2. Bottom insets are close-ups of the regions highlighted with red boxes. Arrows show a cell elongating along the axis roughly perpendicular to the leading edge. Scale bars are 50 µm. N=2.

(**B**) Inverted confocal images of an embryo imaged and annotated as in (A) showing serosal cells stretching orthogonally to the window at Stage 4. Scale bar is 10 µm. N=2.

(**C**) Inverted cartographic projections of a LifeAct-eGFP expressing *Tribolium* embryo imaged and reconstructed with multi-view SPIM. During window closure, highlighted serosal cells increase progressively their shape anisotropy as they leave the leading edge and then become hexagonal inside the tissue.

(**D**) Graph showing the change in anisotropy of cells highlighted in (C) over time normalized to the highest anisotropy for each cell. Anisotropy is defined as deviation from the circle that has circularity value of 1. Time-point 0 is different for each measured cell and corresponds to the stage when it gets evicted from the cable and its anisotropy is the highest.

**Supplementary Figure 3**


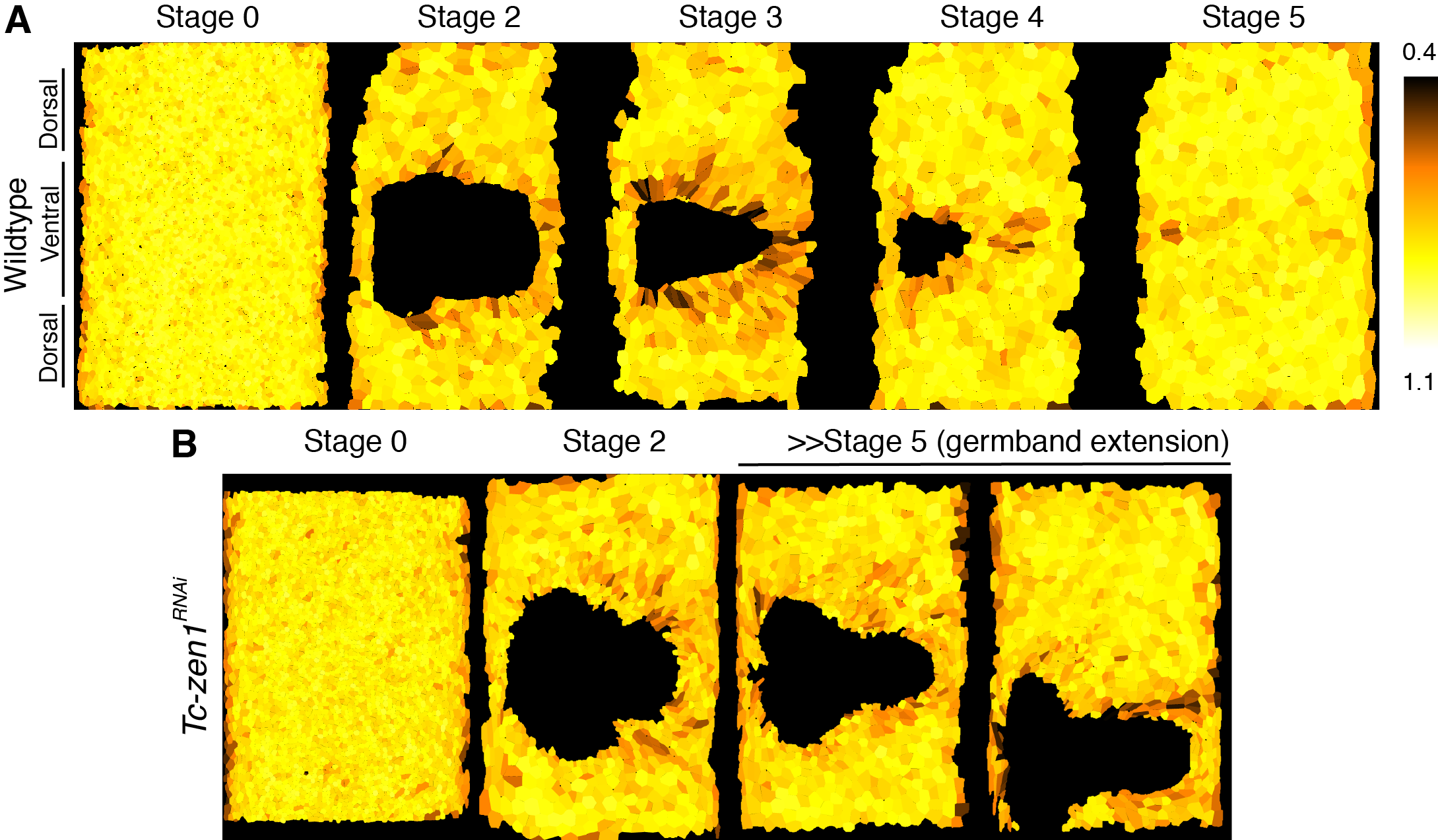


**Supplementary Figure 3:  Cell shape anisotropy in wildtype and *Tc-zen1^RNAi^* embryos**

(**A**) Cartographic projections at reference stages of a wildtype embryo expressing LifeAct-eGFP reconstructed with multi-view SPIM. The projections are overlaid with outlines of serosal cells. Serosal cell in each projection were segmented automatically, curated manually and color-coded according to their anisotropy defined as deviation from the circle that has circularity value of 1.

(**B**) Cartographic projections at reference stages of a *Tc-zen1*^RNAi^ embryo labeled with *Gap43-eYFP* mRNA and reconstructed with multi-view SPIM. Serosal cells in the projections are segmented and color-coded as in (**A**).

**Supplementary Figure 4**

**
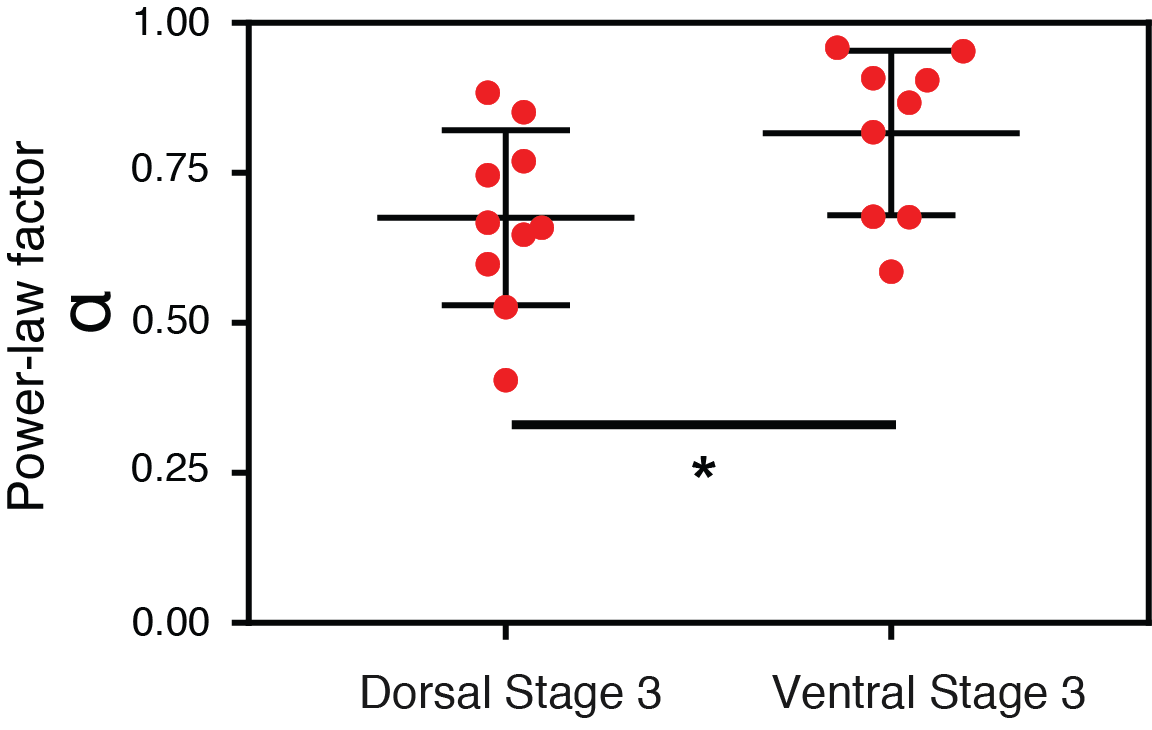
**

**Supplementary Figure 4: Power law analysis of laser ablations**

Distribution of power-law factor α for dorsal and ventral serosa at Stage 3 (see Methods section Laser ablations). Values of α closer to 0 show properties of an elastic solid, while α values closer to 1 indicate viscous fluid properties. The number of embryos (N) used for ablations were as follows: Dorsal ablations N=10; Ventral ablations N=9. Distributions are significantly different at the 0.05 level. P-values between 0.05-0.01 are labelled with *, 0.009-0.001 are labelled with **, <0.001 with ***. Plots indicate the median with a thick line, the mean with a black dot and the standard deviation (s.d.) with the thin error bars.

**Supplementary Figure 5**


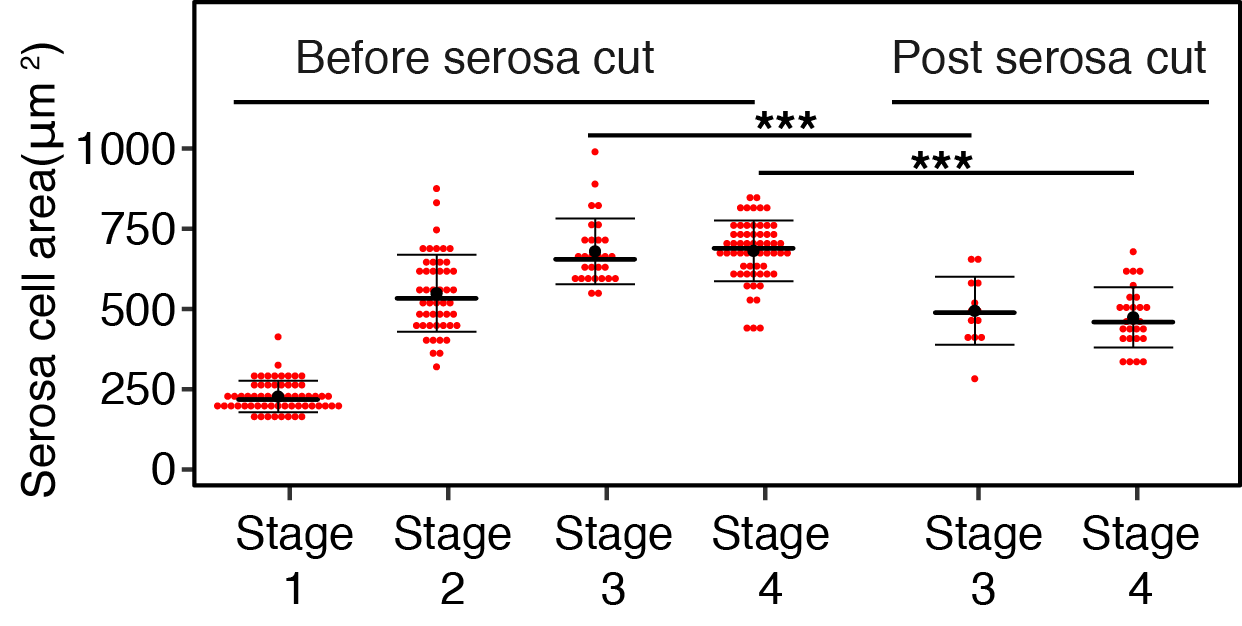


**Supplementary Figure 5: Contraction of cells after release in serosal tension**

Graph showing apical cell areas in the serosa before and after laser ablations at different reference stages. Intact cells neighboring the ablation site in embryos expressing LifeAct-eGFP were measured before and after laser cuts in the dorsal serosa. The number of cells (n) and the number of embryos (N) sampled before serosa cut in the dorsal region: Stage 1 n=116 and N=11; Stage2 n=66 and N=9; Stage 3 n=39 and N=6; Stage 4 n=76 and N=10. Post serosa cut in the dorsal region: Stage 3 n=13 and N=5; Stage 4 n=27 and N=7. P-values between 0.05-0.01 are labeled with *, 0.009-0.001 are labeled with **, <0.001 with ***. Plots indicate the median with a thick line, the mean with a black dot and the standard deviation (s.d.) with the thin error bars.

**Supplementary Figure 6**
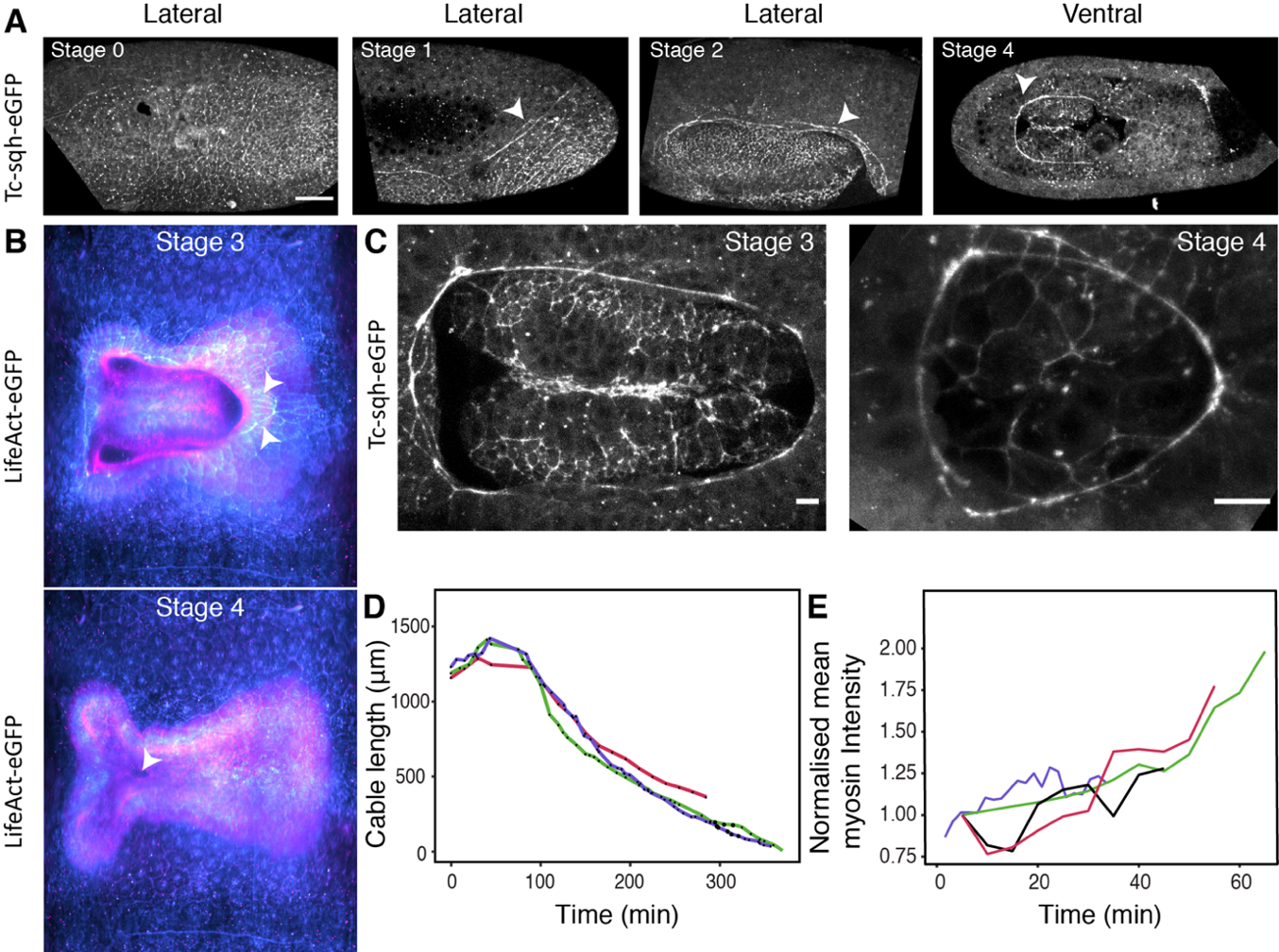


**Supplementary Figure 6:** **Actomyosin cable at the serosa-embryonic boundary**

(**A**) Myosin localization in a *Tribolium* embryos expressing *Tc-sqh-eGFP* and imaged with confocal microscopy. Arrowheads point to the myosin enrichment in a cable-like structure at different stages of serosa epiboly (N=6). Scale bar is 50 µm.

(**B**) Multi-layered cartographic projections of an embryo expressing LifeAct-eGFP reconstructed from a multi-view SPIM recording. Surface layers are shown in blue and internal layers in magenta. The arrowheads point to the cable lining the serosa window posteriorly at Stage 3 and shortly before closure at Stage 4 (N=1).

(**C**) Confocal images of an embryo expressing Tc-sqh-eGFP showing myosin enrichment at the serosal edge during window closure at Stage 3 and Stage 4 (N=6). Scale bars are 10 µm.

(**D**) The graph shows the length of the actomyosin cable as a function of time during serosa epiboly. The cable was manually segmented from cartographic projections of embryos labeled with LifeAct-eGFP or Tc-sqh-eGFP reconstructed from multi-view SPIM recordings (N=3).

(**E**) The graph shows mean myosin intensity normalized to the initial value during serosa window closure. The cable was manually segmented from Tc-sqh-eGFP labeled embryos (N=4) as in (**D**).

**Supplementary Figure 7**


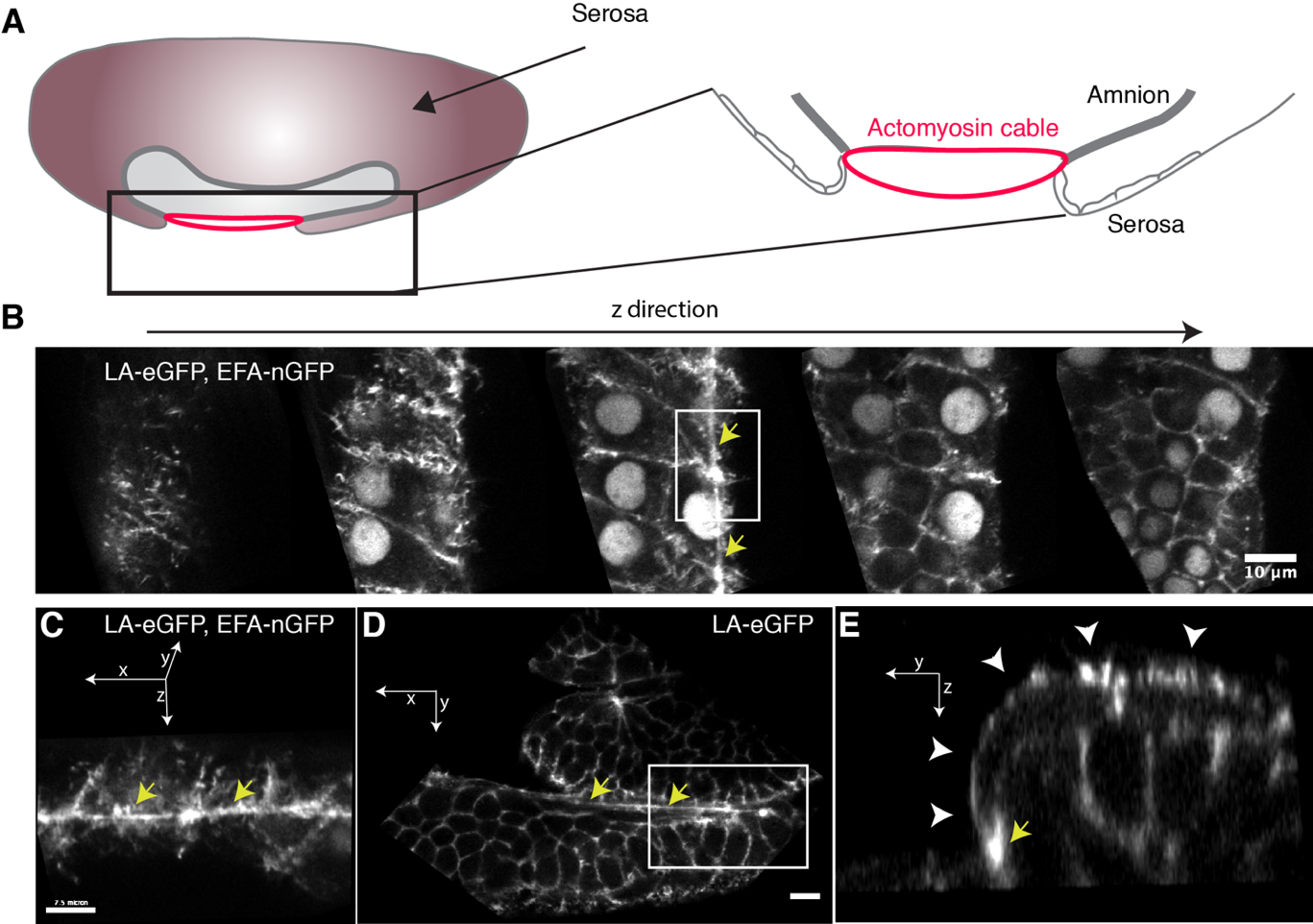


**Supplementary Figure 7:** **Detailed visualization of the actomyosin cable**

**A**) Illustration of a Stage 4 *Tribolium* embryo showing the actomyosin cable lining the serosa window at the serosa/amnion boundary.

(**B**) Slices from a z-stack acquired through a *LifeAct-eGFP*; *EFA-nGFP* transgenic *Tribolium* embryo imaged with confocal microscopy. The arrows in the third slice point to the cable at the serosa window. Scale bar is 10 µm. N=1.

(**C**) Close-up of the region demarcated with the white box in (**B**) orthogonally sliced and rotated to visualize the cable in the middle. Arrows point to the actomyosin cable at the boundary between the serosa (top) and embryonic (bottom) rudiments. Scale bar is 10 µm.

(**D**) Optical slice through an embryo labeled with LifeAct-eGFP showing the actomyosin cable (arrows) at the edge of the serosa window. Scale bar is 10 µm. N=1.

**(E)** Orthogonal view of the region demarcated with the white box in (**D**). Arrowheads indicate the squamous serosa bent inwards over the serosa window and the arrow points to the actomyosin enrichment at the leading edge of the serosa.

**Supplementary Table 1: Overview of imaging datasets**

| Figure | Fluorescent labeling of imaged embryo | xy pixel size (µm) | z step  (µm) | Temporal resolution | Microscope used | Injected/transgenic imaged embryo |
| --- | --- | --- | --- | --- | --- | --- |
| 1C | H2A-eGFP | 0.38 | 2 | 2 min | Zeiss Z1 | Transgenic |
| 1D | nGFP | 0.33 | 2 | 90 sec | Zeiss Z1 | Transgenic |
| 1E | H2A-eGFP OR nGFP OR GAP43-eYFP | 0.38 | 2 | 90 sec | Zeiss Z1 | Transgenic |
| 1F, 2I | LA-eGFP | 0.20 | 1.5 | NA | Zeiss 780 | Transgenic |
| 1G, 2G, 3E | LA-eGFP OR Gap43-eYFP | 0.38 | 2 | 90 sec | Zeiss Z1 | Transgenic, Injected |
| 2B | H2A-eGFP OR nGFP OR LA-eGFP | 0.38 | 2 | 90-120 sec | Zeiss Z1 | Transgenic, Injected |
| 2C, 2E, 2F | LA-eGFP | 0.20 | 1 | 2 min | Zeiss 780 | Transgenic |
| 2D | H2A-eGFP | 0.38 | 2 | 90 sec | Zeiss Z1 | Transgenic |
| 2H | LA-eGFP | 0.38 | 2 | 90 sec | Zeiss Z1 | Injected |
| 3A, 3C | LA-eGFP + nGFP | 0.20 | 1.5 | 2.6 sec | Zeiss 780 | Transgenic |
| 3B, 3D | LA-eGFP | 0.2267 | NA | 0.5 sec | Custom UV ablation setup | Transgenic |
| 4A | Tc-sqh-eGFP | 0.38 | 2 | 5 min | Zeiss Z1 | Injected |
| 4B | Tc-sqh-eGFP, LA-eGFP | 0.38 | 2 | 5 min, 90 sec | Zeiss Z1 | Injected |
| 4C | LA-eGFP | 0.38 | 2 | 90 sec | Zeiss Z1 | Transgenic |
| 4D, 4E, 4F | LA-eGFP | 0.55 OR 0.08 | 2 OR 1.5 | 1.6 sec | Zeiss 780 | Transgenic |
| 4G | LA-eGFP | 0.10 | 1 | 2.5 sec | Zeiss 780 | Transgenic |
| 4H, 4I | Tc-sqh-eGFP | 0.11 | 2 | 14.3 sec | Zeiss 780 | Transgenic |
| 5A | LA-eGFP | 0.47 | 2.5 | 5 min | Zeiss 780 | Transgenic, pupal injection for RNAi |
| 5B | LA-eGFP (WT) | 0.55 | 2 | NA | Zeiss 780 | Transgenic |
|  | LA-eGFP  (Tc-zen1^RNAi^) | 0.33 | 2 | NA | Zeiss 780 | Transgenic, pupal injection for RNAi |
| 5 C, 5H | LA-eGFP | 0.33 | 1 | NA | Zeiss 780 | Injected |
| 5 D, 5I | GAP43-eYFP | 0.20 | 1 | NA | Zeiss 780 | Injected |
| 5E, 5F, 5G, 5J | GAP43-eYFP | 0.38 | 2 | 5 min | Zeiss Z1 | Injected |
