# Supplementary material for: Regionalized tissue fluidization is required for epithelial gap closure during insect gastrulation
Source: Nat Commun. 2020 Nov 5;11:5604. doi: 10.1038/s41467-020-19356-x (PMC7645651; doi:10.1038/s41467-020-19356-x)
Supplement: Supplementary file 2 — Description of Additional Supplementary Files [file 41467_2020_19356_MOESM2_ESM.pdf]

Title: Supplementary Movie 1: Live imaging early *Tribolium* embryogenesis with multi-view SPIM
Description: Lateral (top) and ventral (bottom) views of a transgenic *Histone-eGFP Tribolium* embryo 3D rendered from a multi-view light-sheet recording. The embryo was reconstructed from 5 angles acquired every 1.5 minute at 22°C. Anterior is to the left. Time-stamp is hh:mm:ss.

Title: Supplementary Movie 2: Tissue cartography of *Tribolium* serosa epiboly
Description: Time-lapse cartographic projections generated from a 4D SPIM recording of a transgenic *EFA-nGFP Tribolium* embryo. The expanding serosa is highlighted with changing colors as it increases its area. Anterior is to the left, dorsal at the top and ventral at the bottom. Time-stamp is hh:mm.

Title: Supplementary Movie 3: Tracking of serosal cells participating in window closure
Description: Time-lapse layered cartographic projections generated from a 4D SPIM recording of a transgenic *αTub-H2A-eGFP Tribolium* embryo. The embryo was reconstructed from 5 angles acquired every 1.5 minute. Each frame shows a maximum intensity projection of onion-like cartographic projections of the embryo color-coded by depth to distinguish between superficial serosal (green) and deeper embryonic nuclei (magenta). The nuclei of the last few cells contributing to the serosa window closure are tracked from Stage 1 to Stage 5 and the tracks are color-coded by time. Anterior is to the left, dorsal at the top and ventral at the bottom. Time-stamp is hh:mm.

Title: Supplementary Movie 4: Cell eviction from the leading serosal edge during window closure
Description: Confocal time-lapse imaging showing serosa window closure (Stages 4-5) in a transgenic *LifeAct-eGFP* embryo. The raw frames are shown on the left and selected cells tracked while leaving the serosal edge are highlighted on the right. Ventral view, anterior to the left. Time-stamp is hh:mm. Scale bar is 50 µm.

Title: Supplementary Movie 5: Patterns of neighbor exchange during serosa epiboly
Description: Tracking of abutting rows of serosal cells in time-lapse cartographic projections generated from SPIM imaging of a *Tribolium* embryo injected with *LifeAct-eGFP* mRNA. The same rows of cells are highlighted with colored dots (left half) or with lines (right half). Anterior is to the left, dorsal at the top and ventral at the bottom. Time stamp is hh:mm.

Title: Supplementary Movie 6: Myosin enrichment at the extraembryonic-embryonic boundary
Description: Time-lapse cartographic projections generated from a 4D SPIM recording of an embryo injected with *Tc-sqh-eGFP* mRNA and imaged at 22°C. Myosin intensity is color-coded with the Green-Blue LUT. The dorsal part of the embryo is positioned in the middle and the two ventral halves at the top and the bottom of the cartographic projections. Arrows show the emergence and constriction of the actomyosin cable. Time-stamp is hh:mm.

Title: Supplementary Movie 7: Laser ablation of individual cable-forming cell edges
Description: Time-lapse videos showing the displacement of severed cable-forming cell edges visualized in transgenic *LifeAct-eGFP* embryos. Laser ablations were performed in Stage 1 (left), Stage 3 (middle) and Stage 4 (right) embryos and the edges were tracked with Fiji to measure the recoil velocity over time. Time-stamp is mm:ss.

Title: Supplementary Movie 8: Heterogeneous myosin accumulation in cable-forming cell edges
Description: Confocal time-lapse video of a transgenic *Tc-sqh-eGFP Tribolium* embryo showing serosa window closure during Stage 4. Frames are maximum intensity projections showing myosin intensity with the Green-Blue LUT. Time stamp is mm:ss.

Title: Supplementary Movie 9: Live imaging of a *Tc-zen1*^{RNAi} *Tribolium* embryo
Description: Confocal time-lapse video of a transgenic *LifeAct-eGFP* embryo in which *Tc-zen1* was knocked down using parental RNAi. Lateral view of maximum intensity projections with anterior to the left and ventral to the bottom. Time stamp is hh:mm.

Title: Supplementary Movie 10: Failure of serosa to close in *Tc-zen1*^{RNAi} embryo
Description: Time-lapse cartographic projections of a *Tc-zen1*^{RNAi} *Tribolium* embryo labeled with *GAP43-eYFP* mRNA and reconstructed with 4D SPIM. The ventral part of the embryo is positioned in the middle with anterior to the left. Time stamp is hh:mm.

Title: Supplementary Movie 11: Animation of 3D rendered segmented cartographic maps
Description: Segmented cartographic maps showing serosal cell area (Fig 1G), shape index (Fig 2G), circularity (Supplementary Fig 3), fluidity (Fig 3E) back projected to the original 3D volume and volumetric rendered using Fiji. The edges of the cartographic projections (corresponding to the dorsal midline and poles of the embryos) that were excluded from our quantifications were also excluded from the 3D renderings.
