# Supplementary material for: Regionalized tissue fluidization is required for epithelial gap closure during insect gastrulation
Source: Nat Commun. 2020 Nov 5;11:5604. doi: 10.1038/s41467-020-19356-x (PMC7645651; doi:10.1038/s41467-020-19356-x)
Supplement: Supplementary file 3 — Reporting Summary [file 41467_2020_19356_MOESM3_ESM.pdf]

nature research

Corresponding author(s): Pavel Tomancak, Anastasios Pavlopoulos

Last updated by author(s): August 18th 2020

Reporting Summary

Nature Research wishes to improve the reproducibility of the work that we publish. This form provides structure for consistency and transparency in reporting. For further information on Nature Research policies, see our Editorial Policies and the Editorial Policy Checklist.

Statistics

For all statistical analyses, confirm that the following items are present in the figure legend, table legend, main text, or Methods section.

n/a	Confirmed	
☐	☒	The exact sample size (*n*) for each experimental group/condition, given as a discrete number and unit of measurement
☐	☒	A statement on whether measurements were taken from distinct samples or whether the same sample was measured repeatedly
☐	☒	The statistical test(s) used AND whether they are one- or two-sided<br>*Only common tests should be described solely by name; describe more complex techniques in the Methods section.*
☒	☐	A description of all covariates tested
☐	☒	A description of any assumptions or corrections, such as tests of normality and adjustment for multiple comparisons
☐	☒	A full description of the statistical parameters including central tendency (e.g. means) or other basic estimates (e.g. regression coefficient) AND variation (e.g. standard deviation) or associated estimates of uncertainty (e.g. confidence intervals)
☐	☒	For null hypothesis testing, the test statistic (e.g. *F*, *t*, *r*) with confidence intervals, effect sizes, degrees of freedom and *P* value noted<br>*Give P values as exact values whenever suitable.*
☒	☐	For Bayesian analysis, information on the choice of priors and Markov chain Monte Carlo settings
☒	☐	For hierarchical and complex designs, identification of the appropriate level for tests and full reporting of outcomes
☒	☐	Estimates of effect sizes (e.g. Cohen's *d*, Pearson's *r*), indicating how they were calculated

Our web collection on statistics for biologists contains articles on many of the points above.

Software and code

Policy information about availability of computer code

Data collection	Fiji is a continuous release open source software and it as well as its plugins do not have version numbers.<br>Multiview reconstruction plugin of Fiji available through a Fiji core distribution.<br>Custom Fiji plugins for combining and distortion correction of cartographic projections - available from a dedicated update site https://sites.imagej.net/Ulman2<br>Labkit Fiji plugin for manual curation of segmentation data - available from a dedicated update site https://sites.imagej.net/LabKit<br>StarDist Fiji plugin for machine learning based automated segmentation of cartographic maps - available from a dedicated update site https://sites.imagej.net/StarDist<br>Mastodon Fiji plugin for tracking of cells in large time-lapse sequences - available from a dedicated update site https://sites.imagej.net/Mastodonpreview<br>MaMuT Fiji plugin for tracking of cells in time-lapse sequences - available from a dedicated update site https://sites.imagej.net/MaMuT<br>Toolbox developed by MPI-CBG Scientific Computing Facility for managing segmentations - https://sites.imagej.net/SCF-MPI-CBG<br>Ilastic version 1.1.8 for generating a 3D point cloud of the embryo shape<br>ImSANE software for cartographic projection - https://github.com/idse/imsane. ImSANE does not have a version number<br>Fiji 3D Viewer for rendering - part of core Fiji distribution.
Data analysis	Toolbox developed by MPI-CBG Scientific Computing Facility for manual segementation and tracking of cell outlines in time lapse sequences - available from a dedicated update site https://sites.imagej.net/SCF-MPI-CBG<br>Custom Fiji plugins for measuring cell and tissue areas and extracting shape/alignment indices, anisotropy - available from a dedicated update site https://sites.imagej.net/Ulman2<br>Fiji Manual tracking plugin for following the recoil of tissue edges after laser cut - part of core Fiji installation<br>Fiji ROI manager for calculating cell areas manually - part of core Fiji installation<br>ImageJ Kymograph plugin https://imagej.net/Multi_Kymograph

R version 3.6 and R-studio version 1.2.5001 for all plots

For manuscripts utilizing custom algorithms or software that are central to the research but not yet described in published literature, software must be made available to editors and reviewers. We strongly encourage code deposition in a community repository (e.g. GitHub). See the Nature Research guidelines for submitting code & software for further information.

Data

Policy information about availability of data

All manuscripts must include a data availability statement. This statement should provide the following information, where applicable:

- Accession codes, unique identifiers, or web links for publicly available datasets
- A list of figures that have associated raw data
- A description of any restrictions on data availability

The confocal imaging data and cartographic maps that support the findings of the study are available on Figshare.
Raw light sheet microscopy data will be available on IDR (upload completion pending) or from P.T. upon request. The Figshare and IDR DOIs are listed in Image Datafile.
All data used for generating the plots in the manuscript are provided as a Source Datafile.
All statistics and p-values are reported in the Statistics Datafile.

Field-specific reporting

Please select the one below that is the best fit for your research. If you are not sure, read the appropriate sections before making your selection.

☒ Life sciences ☐ Behavioural & social sciences ☐ Ecological, evolutionary & environmental sciences

For a reference copy of the document with all sections, see nature.com/documents/nr-reporting-summary-flat.pdf

Life sciences study design

All studies must disclose on these points even when the disclosure is negative.

Sample size	All relevant sample sizes are reported in the Figure legends. "N" refers to number of embryos used for calculations and "n" refers to the total number of calculations performed from the same sample embryo. The normal distribution of the data was tested using the Shapiro-Wilk test. Distributions were compared using the non-parametric two-sided Wilcoxon Rank-Sum test or the parametric t-test as applicable.
Data exclusions	For cartographic projections, cells bisected during unfolding of the map at the edges were manually excluded from the analysis and the Figures. The preestablished criterium for exclusion was the disruption of a cell by the need of opening a continuous cylinder along an arbitrarily placed line.
Replication	In all figure legends, independent biological replicates are denoted with capital 'N' and the total number of quantified cells is denoted by lower case 'n'.
Randomization	Most of the observations in this study did not involve comparisons between groups, hence randomization was not relevant. In experiments involving comparisons no particular randomization strategy was implemented.
Blinding	Experiments were evaluated by an objective computational image quantification protocols that cannot be biased. The evaluation of Zen phenotypes compared to wild type could not be blinded because the phenotype is obvious to any annotator. Moreover, map projetions were evaluated by unbiased computational analysis.

Reporting for specific materials, systems and methods

We require information from authors about some types of materials, experimental systems and methods used in many studies. Here, indicate whether each material, system or method listed is relevant to your study. If you are not sure if a list item applies to your research, read the appropriate section before selecting a response.

Materials & experimental systems

n/a	Involved in the study	
☒	☐	Antibodies
☒	☐	Eukaryotic cell lines
☒	☐	Palaeontology and archaeology
☐	☒	Animals and other organisms
☒	☐	Human research participants
☒	☐	Clinical data
☒	☐	Dual use research of concern

Methods

n/a	Involved in the study	
☒	☐	ChIP-seq
☒	☐	Flow cytometry
☒	☐	MRI-based neuroimaging

Animals and other organisms

Policy information about studies involving animals; ARRIVE guidelines recommended for reporting animal research

Laboratory animals	The study used vermillion white strain of Tribolium castaneum. The animals were not segregated based on gender. 1-3 month old adult beetles were used.
Wild animals	No wild animals were used in this study
Field-collected samples	No Field-collected samples were used in this study
Ethics oversight	No ethical approvals were required

Note that full information on the approval of the study protocol must also be provided in the manuscript.
